# Relation between cost of drug treatment and body mass index in people with type 2 diabetes in Latin America

**DOI:** 10.1371/journal.pone.0189755

**Published:** 2017-12-20

**Authors:** Jorge Federico Elgart, Mariana Prestes, Lorena Gonzalez, Enzo Rucci, Juan Jose Gagliardino, for the QUALIDIAB Net study group

**Affiliations:** 1 CENEXA. Center of Experimental and Applied Endocrinology (UNLP-CONICET La Plata), School of Medicine, National University of La Plata, La Plata, Argentina; 2 School of Health Economics and Management of Healthcare Organizations, Faculty of Economic Sciences, National University of La Plata, La Plata, Argentina; 3 III-LIDI, Faculty of Informatics, National University of La Plata, La Plata, Argentina; McMaster University, CANADA

## Abstract

**Aims:**

Despite the frequent association of obesity with type 2 diabetes (T2D), the effect of the former on the cost of drug treatment of the latest has not been specifically addressed. We studied the association of overweight/obesity on the cost of drug treatment of hyperglycemia, hypertension and dyslipidemia in a population with T2D.

**Methods:**

This observational study utilized data from the QUALIDIAB database on 3,099 T2D patients seen in Diabetes Centers in Argentina, Chile, Colombia, Peru, and Venezuela. Data were grouped according to body mass index (BMI) as Normal (18.5≤BMI<25), Overweight (25≤BMI<30), and Obese (BMI≥30). Thereafter, we assessed clinical and metabolic data and cost of drug treatment in each category. Statistical analyses included group comparisons for continuous variables (parametric or non-parametric tests), Chi-square tests for differences between proportions, and multivariable regression analysis to assess the association between BMI and monthly cost of drug treatment.

**Results:**

Although all groups showed comparable degree of glycometabolic control (FBG, HbA1c), we found significant differences in other metabolic control indicators. Total cost of drug treatment of hyperglycemia and associated cardiovascular risk factors (CVRF) increased significantly (p<0.001) with increment of BMI. Hyperglycemia treatment cost showed a significant increase concordant with BMI whereas hypertension and dyslipidemia did not. Despite different values and percentages of increase, this growing cost profile was reproduced in every participating country. BMI significantly and independently affected hyperglycemia treatment cost.

**Conclusions:**

Our study shows for the first time that BMI significantly increases total expenditure on drugs for T2D and its associated CVRF treatment in Latin America.

## Introduction

Obesity represents a large and growing global health problem [1] significantly associated with increased morbidity and mortality [2–5], decreased quality of life [6], and increased healthcare costs [7].

Body fat distribution, particularly excess of visceral adipose tissue (VAT), usually called abdominal obesity (AO), has been associated with greater health risk [8] such as development of type 2 diabetes (T2D), cardiovascular disease (CVD), and serious hospitalization events [9–11].

The Prospective Obesity Cohort of Economic Evaluation and Determinants (PROCEED) study, a multinational observational-prospective internet-based cohort study comparing healthcare utilization of overweight/obese people with and without AO, concluded that more obese people had an increasing gradient of medical conditions, metabolic risk factors, and healthcare utilization [12]. Similar changes were reported by Dilla et al following for a 12-month period 738 patients with a mean age of 66 years and BMI of 30.6 kg/m^2^: each unit gain in BMI was associated with a 20.0% increase in costs for BMI gainers while loss of one unit decreased these costs by 8.0% in non-BMI gainers [13]. Milder et al. also reported that obese persons used more prescription drugs of several types, particularly cardiovascular drugs (OR 3.83 in men and 2.80 in women) and diabetes drugs (OR 5.72 in men and 3.92 in women) than normal weight persons [14]. Future healthcare costs were also higher for overweight persons, especially for those with BMIs ≥ 30 kg/m^2^ [15].

Higher costs of medical treatment associated with obesity have also been observed at primary care level: the Counterweight Project Team reported that total prescribing volume was significantly higher for the group with obesity and increased two- to four fold in the case of drugs such as lipid regulators, β-adrenoreceptor drugs, drugs affecting the renin angiotensin system, calcium channel blockers, sulphonylureas, biguanides and other drugs as well. This increase was due to both the greater number of patients treated and the use of higher drug doses [16,17].

Despite this large and concordant information, Cawley et al suggest that although information on the effect of weight and weight loss on medical expenditures is critical for decision makers to analyze cost-effectiveness of strategies for prevention and treatment of obesity, medical care costs at specific levels of BMI are not well described [18]. Four major problems challenge this assessment: 1) different conditions affect people with and without obesity, and therefore the correlation of medical expenditure with BMI depends not only on the effect of BMI itself but also on the association of other unobserved characteristics; 2) errors in weight and height data due to frequency of self-reporting rather than direct measurements [19,20]; 3) casual association of independent and dependent variables; 4) medical expenditures are a nonlinear function of BMI, and therefore, accurately estimating medical expenditures at various levels of BMI requires a nonlinear model. They concluded that savings from a given percent of reduction in BMI depends on its initial value: the saving is greater the heavier the obese individual is, and is even greater for those with diabetes. Thus, despite the frequent simultaneous presence of T2D and obesity, the specific effect of the latest on the cost of drug treatment of this disease has not been addressed.

To provide an answer to this last issue, we now evaluated the association of overweight/obesity and BMI on the cost of drugs used for treatment of hyperglycemia and associated cardiovascular risk factors (hypertension, dyslipidemia) in people with T2D.

## Research design and methods

### Study population and sampling

This Latin American observational study utilized data from the QUALIDIAB database which includes patients seen at public and private Diabetes Service Centers in Argentina, Chile, Colombia, Peru, and Venezuela. QUALIDIAB is a program that evaluates the quality of care provided to people with diabetes in Latin America. Development of the QUALIDIAB net was based on the benefits of a common data registry in different countries to enable comparison of data to correct mistakes and strengthen successful strategies. The QUALIDIAB database includes clinical, metabolic and therapeutic indicators, information on micro and macrovascular complications, the rate of use of diagnostic and therapeutic elements and annual patient hospitalization [20–22]. All this information is reported directly by physicians during personal interviews; thereafter, data are loaded and stored in anonymous format for subsequent analysis.

We included all patients having filled out a QUALIDIAB form between January 2011 and June 2014. Therefore, records of 4124 patients with T2DM were analyzed. Their country of origin was Argentina (2246), Chile (200) Colombia (654), Peru (651), and Venezuela (373). 1025 records were excluded because we were unable to calculate BMI (due to missing data on weight, height or both); consequently, the final number of people used for the statistical analysis was 3,099.

### Data analysis

BMI was calculated for each participant: weight in kilograms/ (height in meters)^2^. Final BMI data were classified and divided according to the WHO definition into three groups: *Normal weight* (18.5 ≤ BMI < 25), *Overweight* (25 ≤ BMI < 30) and *Obese* (BMI ≥ 30) [1]. Within each category, we utilized clinical and metabolic indicators, as well as type of drug treatment (drug and daily dose prescribed). Drug treatment was classified by drug prescription into three groups (*Hyperglycemia*, *Hypertension* and *Dyslipidemia*).

### Cost of drug treatment calculation

Monthly expenditure on drugs was estimated by micro-costing. For that purpose, we calculated a mean unit retail price per milligram of each drug or per insulin units in each country (except Venezuela). Drug costs were obtained from representative databases in Argentina (Alfabeta.net), Colombia (SISMED), Chile (Kairos@) and Peru (Observatorio de Precios—DIGEMID); and then converted to US dollars ($) according to the exchange rate in August 2014 in each participating country. With these data, we estimated an average price for each drug. Monthly drug treatment expenditures was calculated individually for each patient according to resource utilization, as follows: the daily dose was multiplied by 30 (a month), then multiplied by the average price, resulting in monthly expenditures. Since the average price of each drug used was the same for different settings and countries, drug treatment cost reflects only the extent of drug utilization.

### Statistical analysis

Statistical analyses utilized the Statistical Package for Social Sciences version 15 (SPSS Inc, Chicago, IL, US). Descriptive statistics are presented as percentages and mean ± standard deviation (SD). Group comparisons for continuous variables utilized parametric or nonparametric tests according to the data distribution profile. The Chi-square test was used to estimate differences between proportions. Multivariable regression analysis was used to evaluate the association between cost of drug treatment and BMI. For regression analysis, to account for the skewed distribution of cost of drug treatment we developed a generalized linear model (GLM) with log-link function to estimate the association between drug treatment cost and BMI, patient demographic characteristics, diabetes treatments, complications and comorbidities. When several independent variables were highly correlated with each other (correlation coefficient ≥0.25), only 1 variable was included in the model. For example, age and diabetes duration are highly correlated, so age was deleted from the models. While we did not consider interaction effects for our analyses, the level of significance was established as p≤0.05.

### Ethical statement

The study protocol was analyzed and approved by the Bioethical Committee of the National University of La Plata. This study was developed according to Good Practice Recommendations (International Harmonisation Conference) and the ethical guidelines of the Helsinki Declaration. Informed consent was waived because this retrospective study involves secondary analysis of existing database which was de-identified and anonymously stored to protect private information. Therefore, this procedure ensured compliance with the National Law 25.326 of Personal Data Protection.

## Results

Clinical and metabolic characteristics of the study population classified according to its BMI showed that only 16% of participants had BMI within the normal range, 38% were overweight, and the remaining 46% were obese (Table 1). The percentage of men was significantly greater in the overweight group than in the other two groups. Mean age was comparable in the normal and overweight groups but was significantly lower (younger) in the obese group. Duration of diabetes was lower in obese than overweight group, while was comparable in the normal and overweight groups. Although systolic and diastolic blood pressure values were within normal range in all groups, they were significantly lower in the normal weight compared to the obese group.

Fasting blood glucose (FBG) and hemoglobin A1c (HbA1c) showed comparable values in all groups; however, they were all above those recommended by the American Diabetes Association and European Association for the Study of Diabetes (ADA/EASD) guideline [23].i.e. all studied population had a comparable degree of abnormal glycometabolic control.

**Table 1:** Characteristics of the study population.

Parameter	All		Normal weight (18.5 ≤ BMI < 25)		Overweight (25 ≤ BMI < 30)		Obesity (BMI ≥ 30)	
	Value	n	Value	n	Value	n	Value	n
Men (%)	42.3	3099	40.0	495	47.1 *	1185	39.1 †	1419
Age (years)	62.1 ± 11.8	3079	63.9 ± 12.4	489	63.3 ± 11.7	1179	60.4 ± 11.4*†	1411
Diabetes Duration (years)	10.8 ± 9.0	2391	11.0 ± 9.7	387	11.5 ± 9.4	896	10.1 ± 8.4 †	1108
BMI (kg/m^2^)	30.1 ± 5.8	3099	23.0 ± 1.4	495	27.4 ± 1.4*	1185	34.9 ± 4.9*†	1419
SBP (mmHg)	129.9 ± 17.5	3053	124.8 ± 18.1	486	129.8 ± 17.5 *	1170	131.7 ± 16.9*†	1397
DBP (mmHg)	77.3 ± 10.9	3049	73.1 ± 10.9	486	76.5 ± 10.3 *	1168	79.3 ± 10.9 *†	1395
FBG (mg/dL)	152.3 ± 84.0	2842	158.2 ± 115.3	441	148.7 ± 76.6	1073	153.3 ± 77.1	1327
HbA1c (%)	7.7 ± 1.9	2739	7.7 ± 2.3	440	7.7 ± 1.8	1032	7.7 ± 1.8	1267
*[mmol/mol]*	*[61 ± 20*.*8]*		*[61 ± 25*.*1]*		*[61 ± 19*.*7]*		*[61 ± 19*.*7]*	
HbA1c ≤7% (%)	43.9	2739	48.2	440	43.1	1032	43.0	*1267*
Total Cholesterol (mg/dL)	185.6 ± 45.9	2749	181.2 ± 45.2	422	185.6 ± 44.7	1050	187.2 ± 46.9	1277
HDL-c (mg/dL)	47.4 ± 21.1	2509	51.3 ± 29.1	393	46.9 ± 14.7 *	946	46.6 ± 22.2 *	1170
LDL-c (mg/dL)	106.7 ± 35.9	2413	104.6 ± 38.4	373	107.3 ± 35.6	918	106.9 ± 35.5	1122
Triglyceride (mg/dL)	171.6 ± 108.4	2671	143.5 ± 87.8	412	162.0 ± 100.2 *	1002	188.4 ± 117.7 *†	1257
Complications (%)	56.1%	2539	59.5%	395	56.3%	969	54.8%	1175
Hypertention (%)	65.3%	2024	53.7%	266	63.3% *	750	71.0% *†	1008
Dyslipemia (%)	62.6%	1939	60.4%	299	59.4%	704	66.0% †	936

Each value represents mean ± SD (standard deviation); BMI: body mass index; SBP: systolic blood pressure; DBP: diastolic blood pressure; FBG: fasting blood glucose.

*Significant compared with normal weight (P < 0.05)

† Significant compared to overweight (p < 0.05).

Triglyceride values were above those recommended by international guidelines in the overweight and obese group, being significantly higher in the latter.

Significant differences between groups were found in hyperglycemia treatment (Table 2). The proportion of patients treated with only diet and physical activity as well as those on oral monotherapy decreased significantly in overweight/obese people, whereas administration of combined oral therapy alone or associated with insulin was significantly higher in the overweight and obese groups. No significant differences among groups were recorded in people treated only with insulin.

**Table 2:** Type of hyperglycemia medications treatment *by BMI categories*.

Treatment	All		Normal weight (18.5 ≤ BMI < 25)		Overweight (25 ≤ BMI < 30)		Obesity (BMI ≥ 30)	
	%	n	%	n	%	n	%	n
**Only diet and Phys. activity**	**2.9**	**91**	**5.2**	**26**	3.2*	**38**	1.9 *†	**27**
**OAD Monotherapy**	**29.9**	**928**	**35.4**	**175**	29.0*	**344**	28.8*	**409**
*Metformin*	*84*.*5*	*770*	*75*.*0*	*132*	*82*.8*	*284*	*90*.3*†	*354*
*SU*	*10*.*3*	*94*	*14*.*8*	*26*	*12*.*2*	*42*	*6*.6*†	*26*
*DPP-4*	*4*.*5*	*41*	*10*.*2*	*18*	*4*.1*	*14*	*2*.3*	*9*
*Other (%)*	*0*.*7*	*6*	*0*.*0*	*0*	*0*.*9*	*3*	*0*.*8*	*3*
**Combined OAD (2 or more)**	**29.0**	**898**	**24.8**	**123**	**29.5**	**350**	**30.0**	**425**
*Metformin (%)*	*97*.*6*	*898*	*96*.*0*	*119*	*97*.*7*	*344*	*98*.*0*	*435*
*SU (%)*	*74*.*9*	*689*	*73*.*4*	*91*	*75*.*9*	*267*	*74*.*5*	*331*
*DPP-4 (%)*	*32*.*0%*	*294*	*35*.*5*	*44*	*32*.*4*	*114*	*30*.*6*	*136*
*Other (%)*	*8*.*2*	*75*	*1*.*6*	*2*	*4*.*5*	*16*	*12*.8*†	*57*
**Insulin + OAD (1 or more)**	**23.1**	**715**	**17.6**	**87**	22.3 *	**264**	25.6 *†	**364**
**Insulin alone**	**15.1**	**467**	**17.0**	**84**	**16.0**	**189**	**13.7**	**194**

OAD: Oral antidibetics drugs; SU: sulfonylureas; DPP-4: dipeptidil peptidasa-4.

*Significant compared with normal weight (P < 0.05)

† Significant compared to overweight (p < 0.05).

Total monthly *per capita* costs of drug treatment of hyperglycemia and associated cardiovascular risk factors increased significantly with BMI category (p<0.001, Table 3). While drug treatment of hyperglycemia alone showed a similar increase trend and percentage, no significant differences were observed in hypertension and dyslipidemia drug therapy.

**Table 3:** Monthly cost of drug treatment *per capita by risk factor and BMI categories*.

Treatment	Normal weight (18.5 ≤ BMI < 25)		Overweight (25 ≤ BMI < 30)			Obesity (BMI ≥ 30)			p value (between groups) ^b^
	Mean ± SD	n	Mean ± SD	Ratio^a^	n	Mean ± SD	Ratio^a^	n	
Hyperglycemia	$61.0 ± 55.4	469	$69.6 ± 57.7 *	1.14	1147	$84.5 ± 76.3 *†	1.38	1392	<0.001
Hypertension	$40.1 ± 30.1	232	$42.7 ± 33.3	1.06	666	$44.4 ± 37.6	1.11	908	0.289
Dyslipidemia	$73.1 ± 36.8	234	$74.2 ± 41.8	1.02	534	$70.0 ± 39.5	0.96	702	0.088
Total cost (All together)	$113.8 ± 83.8	495	$128.2 ± 89.3 *	1,13	1185	$151.2 ± 109.4 *†	1.33	1419	<0.001

Costs of treatment are expressed in US dollars ($).

^a^Based on Normal weight.

^b^ Kruskall-Wallis test.

*Significant compared with Normal weight (p< 0.05; Mann-Whitney U test)

† Significant compared to Overweight (p< 0.05; Mann-Whitney U test).

Even with a comparable degree of glycometabolic control, overweight and obese people increased their monthly *per capita* cost of hyperglycemia medications by 14 and 38%, respectively (Table 3). Each year, overweight and obese people spent, respectively, US$172.80 and US$448.80 more than normal weight patients.

**Fig 1:**
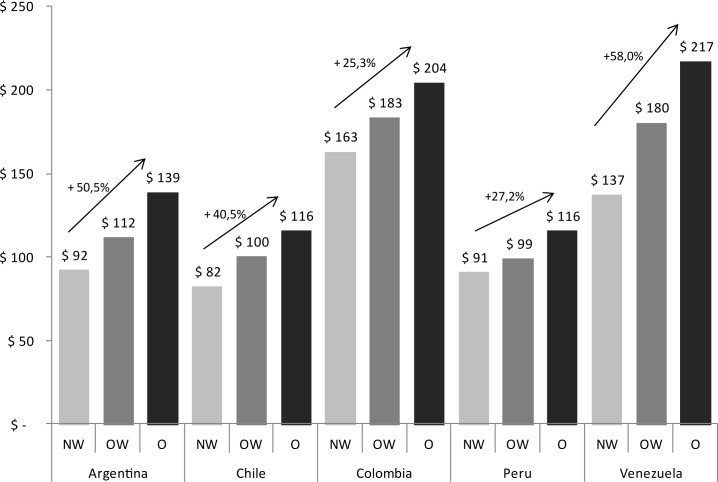
Total cost of drug treatment by country. NW: Normal weight (18.5 ≤ BMI < 25); OW: Overweight (25 ≤ BMI < 30); O: Obesity (BMI ≥ 30).

Since the study includes different numbers of participants from each country, we tested whether BMI and total drug treatment cost showed similar relationship or merely reflected results in a particular country with a larger representation. Although the values differed and the percentage of increase varied, the growing cost profile was reproduced in every participating country (Fig 1). In this regard, the largest and lowest increases were recorded in Venezuela and Colombia, respectively. Since the average price of each drug utilized was the same in the different countries, these differences might be probably ascribed, at least partly, to the quantity of drug utilization.

Multivariable regression analysis showed that total expenditure of drugs was significant and independently associated with gender, BMI, systolic blood pressure (SBP) and LDL-cholesterol, levels, hypertension, dyslipidemia and treatment of T2D (Table 4). Total expenditure on drugs was 7.3% higher in male than in female while total drugs treatment costs were higher in patients with hypertension or dyslipidemia (1.417 and 2.077, respectively). Further, each point of change in BMI was associated with a 1.3% increase in the total drugs expenditure. The analysis also showed that expenditure for hyperglycemia drugs treatment was significantly associated with gender, duration of diabetes, BMI and LDL-cholesterol values, dyslipidemia, T2D complications and type of drugs employed.

**Table 4:** Multivariable regression analysis.

Explanatory Variable	Model 1					Model 2				
	Total cost of drug treatment					Cost of hyperglycemia drug treatment only				
	Original regression coefficient	p value	Exponential ^a^	95% CI		Original regression coefficient	p value	Exponential ^a^	95% CI	
				Lower	Upper				Lower	Upper
Intercept	3.310	0.000	27.397	19.959	37.607	3.333	0.000	28.012	18.916	41.481
Gender (male)	0.070	0.030	1.073	1.007	1.143	0.095	0.017	1.100	1.017	1.189
Diabetes Duration (years)	0.003	0.161	1.003	0.999	1.007	0.013	0.000	1.013	1.008	1.018
HbA1c (%)	0.016	0.099	1.017	0.997	1.036	0.020	0.101	1.020	0.996	1.044
BMI (kg/m2)	0.012	0.000	1.012	1.006	1.018	0.022	0.000	1.022	1.015	1.029
SBP (mmHg)	0.002	0.019	1.002	1.000	1.004	-0.001	0.379	0.999	0.997	1.001
LDL- Cholesterol (mg/dL)	-0.001	0.016	0.999	0.998	1.000	-0.002	0.000	0.998	0.997	0.999
Triglyceride (mg/dL)	0.000	0.194	1.000	1.000	1.001	0.000	0.978	1.000	1.000	1.000
***Hypertension***										
No (Reference)	0.000		1.000					1.000		
Yes	0.349	0.000	1.417	1.318	1.525	0.001	0.987	1.001	0.915	1.094
***Dyslipidemia***										
No (Reference)	0.000		1.000					1.000		
Yes	0.731	0.000	2.077	1.938	2.225	0.224	0.000	1.251	1.149	1.362
***T2D Complications***										
None (reference)	0.000		1.000					1.000		
Micro or Macrovascular	-0.035	0.301	0.966	0.903	1.032	-0.160	0.000	0.852	0.785	0.925
***Treatment of T2D***										
Not using insulin (reference)	0.000		1.000					1.000		
Using Insulin	0.338	0.000	1.402	1.307	1.505	0.573	0.000	1.774	1.628	1.933

^a^ Exp (original regression coefficient).

95% CI: 95% Confidence Interval. BMI: body mass index; SBP: systolic blood pressure; DBP: diastolic blood pressure.

## Discussion

Our data show that increase in BMI is associated with a parallel growth in overall drugs treatment cost of hyperglycemia and its associated cardiovascular risk factors. This effect was observed despite comparable values for FBG/HbA1c and blood pressure found in each BMI classified group. However, impaired serum lipid profile, characterized by significantly high triglyceride level, was recorded mainly in the overweight/obese groups. Based on these results and the fact that treatment costs were expressed *per capita*, we could assume that people with higher BMI required larger quantities of drugs to attain a given blood glucose treatment target. This larger drug demand could be due to the negative impact of overweight/obesity on tissue-insulin sensitivity [24].

Other authors have reported an incremental impact of overweight/obesity on health care costs for people with diabetes. Yu et al found that in patients with diabetes who gained a minimum of one pound between two weight measurements, the average 1-year total diabetes-related health care cost following the second weight measure was significantly higher than the corresponding measure for those who did not gain weight ($2,141 *vs*. $1,869, respectively; p = 0.006). When weight gain and no gain were modeled separately, 1% weight loss was associated with a 5.8% ($131; p<0.01) decrease in diabetes-related cost; the economic benefit of weight loss was higher in the group with BMI ≥ 30 kg/m^2^ [25]. Similarly, Apovian et al reported that obesity is associated with a more than 13-fold increase in expenditure on antidiabetic medications [26].

Despite differences in the magnitude of the cost increase, similar trends were reported regarding the association of BMI on general drug consumption at every level of care complexity [16,17,27,28] and in different health care settings [29–31]. Further, both old and new reports [3,15] as well as current studies of care costs have shown the same trend [7,15].

Although with different values, we found similar association between BMI and medication costs in each participating country (Fig 1). Since we cannot assure that overweight/obesity frequency was exactly the same in all of them, this potential difference could be one of the causes for the different cost increase among countries. However, the persistence of a significant difference in every country -large despite different values- plays in favor of the strength of this phenomenon.

Higher BMI is also associated with higher indirect costs and lower productivity, consequently, its negative impact affects not only health care costs but also many other social factors [27,32,33].

Despite the known negative effects of overweight/obesity associated with T2D on health care costs, productivity, and society, plus the availability of effective strategies for its prevention and treatment [34,35], both obesity and T2D show progressive growth worldwide, reaching epidemic levels [36].

Behavioral treatment of obesity at the primary care level including monthly counseling visits and a choice of meal replacements or weight loss medication could be a cost-effective strategy for obesity over the long term (≥ 10 years) [37]. More aggressive strategies such as bariatric surgery, when appropriately prescribed, may lead to significant cost savings for health care systems [38]. In fact, people with T2D who undergo bariatric surgery may also decrease drug treatment costs compared to those in conventional treatment ($14,346 *vs*. $19,511; p<0.0001) [39]. Further, NICE guideline recommends bariatric surgery as a clinically- and cost-effective option for obese patients with T2D, particularly those with severe obesity [40]. Altogether, all these data suggests that implementation of a large scale weight loss program could effectively decrease this multisectorial disease burden.

If we consider that overweight and obesity: a) increased the monthly *per capita* cost of hyperglycemia drug treatment by 14 and 38%, respectively; b) result in an annual expenditure of $172.80 and $448.80, respectively, higher than for T2D patients with normal weight in order to reach comparable degrees of glycometabolic control and c) overweight/obese people represent 84% of the Latin American T2D population, these three conditions clearly impose a heavy burden on the health care budget. Therefore, this situation should alert health policy makers on the importance of implement effective strategies to reduce overweight and obesity in people with T2D.

Although clear and significant, our results should be considered with caution due to several limitations such as a) it is an observational rather than a prospective study; b) the population studied was not entirely representative of a population base; c) its total number of cases is strongly influenced by one participating country (Argentina) and d) we have not considered neither the physical activity load nor adherence to healthy meal plan thus, we do not know whether such conditions could affect all the BMI conditions in a comparable manner; e) data were reported by specialized diabetes facilities. However, we have shown that the cost profile was comparable in every participating country, and other authors have also shown that in different countries the impact of BMI on drug-treatment costs occurs even at the primary care level [16,17]. In this context, our results seem to reflect a general process rather than local or other bias.

## Conclusions

In brief, our study shows a significant association between BMI and the total cost of drug treatment for T2D and its associated cardiovascular risk factors in Latin America. They also suggest that implementation of effective obesity preventive strategies might significantly decrease the burden of T2D on healthcare costs and other social factors.
